# Chronic Non-specific Low Back Pain and Motor Control During Gait

**DOI:** 10.3389/fpsyg.2018.02236

**Published:** 2018-11-23

**Authors:** Cathrin Koch, Frank Hänsel

**Affiliations:** Institute of Sport Sciences, Technische Universität Darmstadt, Darmstadt, Germany

**Keywords:** non-specific low back pain, motor control, gait, walking, ROM, EMG, kinematics

## Abstract

**Background:** Chronic non-specific low back pain (LBP) poses a major socioeconomic problem, although the mechanisms are not yet clear. Impaired motor control is one of the mechanisms being discussed.

**Objectives:** The purpose of this review is to provide an overview of motor control parameter differences between individuals with and without non-specific LBP during gait.

**Methods:** A literature search on Medline, SportDiscus, PsychInfo, PsychArticels, EMBASE, and Scopus was performed. Twenty-nine articles comparing healthy adults and adults with chronic non-specific LBP in neuromuscular and/or biomechanical parameters during walking or running were examined. Data extraction and quality assessment were independently performed by two persons. Among others, we extracted population, conditions, outcome measures, and results.

**Results:** The results showed that persons with and without non-specific LBP differed in several parameters of motor control, which was indicated by a lower movement amplitude of the pelvis, more in-phase coordination, lower ground reaction forces, higher stride-to-stride variability and a higher activity in ES in the LBP group.

**Conclusion:** Despite no strong evidence for any of the parameters, a combination of biomechanical and neuromuscular parameters provides a conclusive explanation. Impaired motor control during walking is reflected in higher activity of the erector spinae, which leads to a stiffened lumbar-pelvic region. Different acquisition and processing of data renders making comparisons difficult, whereby standards for future research are necessary.

## Introduction

Chronic low back pain (LBP) causes high costs, whereby it presents a socioeconomic burden (Dagenais et al., 2008). For about 85% of back pain, no specific cause of pain—like structural changes or inflammation—can be identified. This is why it is referred to as non-specific back pain (O'Sullivan, 2005). The number of people who need treatment due to back pain is high, although its causes remains unclear. A variety of mechanisms for unspecific back pain are discussed (Saragiotto et al., 2016), including the notion that impaired motor control could possibly be one of the reasons (Götze et al., 2015; Saragiotto et al., 2016). Altered activity patterns of abdominal and extensor muscles (Ghamkhar and Kahlaee, 2015), a restricted range of motion (Laird et al., 2014), and disturbed proprioception (Radebold et al., 2001) are the parameters indicating that motor control is disturbed in patients with chronic LBP. Changes in the neuromuscular system affect movement due to the connection between the neuromuscular activity and biomechanical consequences. For example, higher muscle activity results in slower movements and a reduced range of motion (van Dieën et al., 2003).

Impaired motor control affects movements of everyday life. Given that walking is one of the most common activities, in this review we chose walking to examine the restriction of motor control in patients with chronic LBP regarding activation patterns and resulting biomechanics. Hodges and Tucker (2011) proposed that changes take place at multiple levels of the motors system. A redistribution of activity within and between muscles is associated with changes of the mechanical behavior. Altered movement patterns can occur through pain, injury or instability. They serve as a protection strategy in the short term, although have long-term consequences since they remain even if the actual cause is resolved (Hodges and Tucker, 2011).

We found only a few recently-published reviews considering differences in motor control in persons with and without back pain. One review by Ghamkhar and Kahlaee (2015) considers motor control during walking, although it only investigates muscle activation patterns during gait in people with and without chronic LBP. No review summarizing differences between groups in kinematic gait pattern to gain a full impression of what the changes in motor control during gait in LBP are can be found.

The main question of our review is: What are the differences in motor control between persons with and without chronic non-specific LBP in gait? In order to investigate these differences, we specifically considered case control studies comparing people with and without chronic non-specific LBP. The purpose of this review is to provide a systematic overview of changes at the biomechanical and neuromuscular level. Regarding the growing number of studies on motor control in individuals with and without LBP in walking, we want to collate data to provide an evidence base for future therapeutic interventions.

## Methods

The review has been written according to the guideline of the PRISMA checklist (Liberati et al., 2009).

### Search strategy

**Figure 1:**
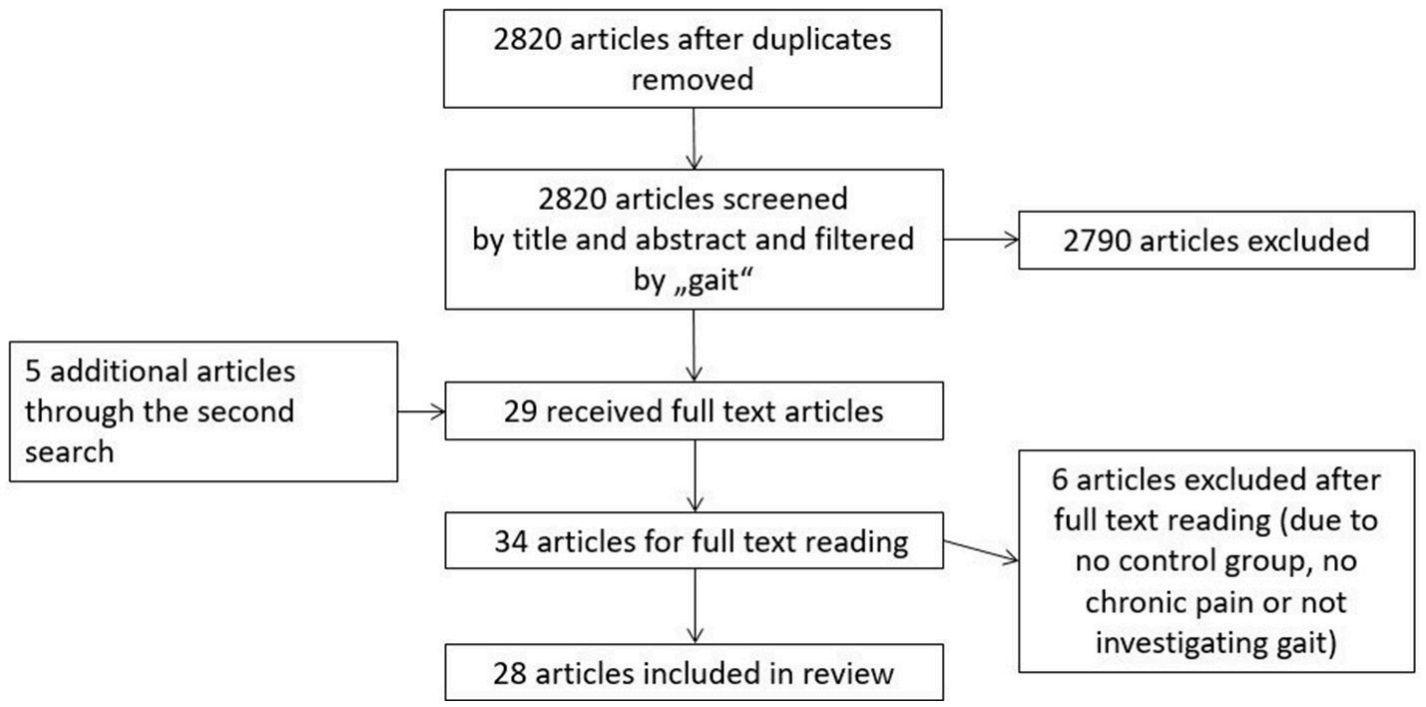
Presenting a flow chart of search strategy and study selection.

First, five databases (Medline, SportDiscus, PsychInfo, PsychArticels, EMBASE, Scopus) were searched for papers published between January 2000 and January 2018 in February 2018. “Low back pain” OR “lumbar pain” AND (“motor control” OR “coordination” OR “movement disorder” OR “variability” OR “stability” OR “proprioception” OR “muscle activation” OR “electromyography” OR “kinematics” OR “center of pressure” OR “range of motion” OR “muscle activity”) Not (“invasive” OR “spinal stenosis” OR “injury” OR “case study” OR “disc herniation” OR “fractures” OR “amputation” OR “taping” OR “strength” OR “metabolic”) were the keywords in a subject term search. A second search was conducted on the same databases by using “low back pain” OR “lumbar pain” in combination with either “gait,” “walking” or “running” as keywords to spread the search. Reference lists of included studies were also scanned to find additional studies. The search strategy was limited to articles written in English and German. The search strategy is also represented in Figure 1.

### Study selection

Eligible studies were screened by title and abstract according to inclusion and exclusion criteria. Articles were included if they studied persons with chronic non-specific LBP compared to healthy controls in any parameter of motor control during walking/running. Exclusion criteria were investigation of professional athletes, physiotherapeutic interventions, psychological interventions, specific back pain (e.g., with a diagnosis of herniated disk or back pain due to injuries, pregnancy, or amputation), operation, intake of medicine, studies without a control group, studies testing quality criteria of a system and reviews.

### Methodological quality

Differences in methodological quality could be one reason for the disparities in the results of the studies. Therefore, the quality of each study was assessed. In order to assess quality, we used a modified version of the Newcastle-Ottawa-Scale (NOS) (Wells et al., 2000). NOS is commonly used as a quality assessment tool in case control studies. Furthermore, it is evaluated as appropriate and easy to use by Deeks et al. (2003). Based on the recommendation of this review, we decided to add three questions from the Quality Assessment Tool for Quantitative Studies of the Effective Public Health Practice Project (Thomas, 2003) to the scale. We added questions about the methods of data acquisition. The applied assessment tool comprised ten questions divided into three categories: 1. selection of cases and controls, 2. comparability and 3. data acquisition (see Supplementary Material *quality assessment score*). The ten criteria for the assessment of the methodological quality were scored as positive (“yes”), negative (“no”) or unclear (“not reported”). Each criterion that was scored positive contributed one or two points to the summary quality score. There were two criteria where two points could be awarded. For the calculation of the score, we did not differentiate between negative or unclear answers. A maximum of 12 points could be scored. Quality assessment was independently performed by two persons. Differences were discussed and—if necessary—disparities were resolved by a third person.

The assessment of evidence was performed in analogy with Van Tulder et al. (2003). In order to evaluate evidence as high consistent results (>75%) from high quality studies are necessary. We evaluated evidence as moderate if one high quality study and/or more studies of moderate quality show consistent results. There is conflicting evidence if the results are inconsistent. Furthermore, we decided to add “tendency” as a category when a slight majority of studies (between >55% and <75%) with higher average quality report the same results.

### Data extraction

Data extraction was independently performed by two persons. Purpose, study population, inclusion/exclusion criteria, design, equipment, outcome measures, and findings were extracted from the full text version of the articles. Articles were divided into two sub-categories according to the type of parameter (biomechanical and neuromuscular).

## Results

### Literature search

A total of 2,820 articles were identified in the search after duplicates had been removed. These articles were screened by title and abstract according to inclusion and exclusion criteria and regarding the content of walking/running. This strategy of sorting by title and abstract according to gait was to ensure that fitting articles were not rejected due to a strong variety of search terms. A second search with search terms as described above was completed. Another five articles could be identified in the same databases. Moreover, reference lists of included articles were considered to avoid missing any articles. After reading the full text articles, we had to exclude three articles, since they were lacking a control group or not investigating gait. Twenty-eight articles about chronic back pain and motor control in walking or running were included in this systematic review.

### Classification of studies

In all included studies, 841 subjects were investigated (LBP: 467, asymptomatic: 374). Sample sizes varied from 4 (Poosapadi Arjunan et al., 2010) to 59 (van der Hulst et al., 2010a) patients who had chronic back pain for at least more than 3 months to chronic pain with for more than 8 months (Hanada et al., 2011). In five studies, only one gender was included. In one study, participants were only female (Zahraee et al., 2014), whereas in three (Vogt et al., 2001; Poosapadi Arjunan et al., 2010; Prins et al., 2016) only men were tested. Further details of study characteristics are described in Supplementary Table 1. The Visual Analog Scale (VAS) (six studies) and Numeric Rating Scale (six studies) were used to quantify the severity of pain. The Ostwestry Disability Index (ODI) (ten studies) and Roland-Morris Questionnaire (six studies) were used to classify disability.

Various parameters were calculated from ground reaction force data, EMG data and motion analysis data. In EMG data EMG activity, EMG variability as well as onset times were the main parameters examined. Motion data were used to calculate preferred walking velocity, stride length and duration, ground reaction forces (GRF), movement amplitude and pattern of gait coordination.

### Quality of studies

A maximum of twelve points could be achieved in quality score. A score of eight and higher was defined as high quality. As shown in Supplementary Table 2, nine points was the highest achieved score (van der Hulst et al., 2010a,b; Ebrahimi et al., 2017). A further three studies are of high quality with a score of eight (Vogt et al., 2003; Zahraee et al., 2014; Gombatto et al., 2015). Another twelve articles (Selles et al., 2001; Vogt et al., 2001; Lamoth et al., 2006a,b; Lee et al., 2007; Newell and van der Laan, 2010; Poosapadi Arjunan et al., 2010; Seay et al., 2011a; Pakzad et al., 2016; Prins et al., 2016; Christe et al., 2017; Kim et al., 2017) scored seven or six points. Ten papers (Lamoth et al., 2002; Hanada et al., 2011; Seay et al., 2011b, 2014; van den Hoorn et al., 2012; Crosbie et al., 2013; Hamacher et al., 2014, 2016; Müller et al., 2015; Manciopi et al., 2017) showed limitations in quality since they did not use a matched group design or clearly define their sample. It is striking that two criteria were not reported in any of the studies. None of the studies show whether outcome assessors were aware of the exposure status of the participants (question eight). A non-response rate was not reported in any of the studies either (question ten).

### Synthesis of results

The results were organized in the categories of biomechanical and neuromuscular data. At the beginning of each paragraph, we present the results. Subsequently, confounders like gait conditions, participants' age and chronicity of pain were taken into consideration to find reasons for inconsistency.

#### Results of biomechanical data

One often-examined biomechanical parameter is preferred walking speed. We found seven out of 13 studies (Selles et al., 2001; Lamoth et al., 2002, 2006a,b; Lee et al., 2007; van den Hoorn et al., 2012; Müller et al., 2015) reporting a lower walking speed in LBP group compared to the control group. On the other hand, there are also six studies that did not find any differences in walking velocity between groups (Newell and van der Laan, 2010; Hanada et al., 2011; Seay et al., 2011a; Zahraee et al., 2014; Christe et al., 2017; Ebrahimi et al., 2017). Two of these studies not finding a difference had high quality (Zahraee et al., 2014; Ebrahimi et al., 2017). Six out of the 13 studies (Selles et al., 2001; Lamoth et al., 2002, 2006a,b; Newell and van der Laan, 2010; van den Hoorn et al., 2012) performed their walking trails on a treadmill, while the others used walkways. Thus, the studies using a treadmill—with one exception—all found differences in walking velocity. Thus, walking condition seem to affect the results. There was no systematical difference in the average age of LBP patients and the duration of chronic pain. Since the majority of studies reported different results than two high quality studies, we see conflicting evidence overall. However, when walking on a treadmill, the results provide a hint of differences between groups.

We also found seven studies examining stride length, two of which (Lamoth et al., 2006a,b) showed a shorter stride length in LBP group at least if participants walked at certain speed. Five other studies did not find any difference in stride length between groups (Newell and van der Laan, 2010; van den Hoorn et al., 2012; Zahraee et al., 2014; Gombatto et al., 2015; Müller et al., 2015). Two of these studies had high quality (Zahraee et al., 2014; Gombatto et al., 2015). Four studies performed their test on a treadmill (Lamoth et al., 2006a,b; Newell and van der Laan, 2010; van den Hoorn et al., 2012), while the other three used a walkway for investigation stride length in preferred speed (Zahraee et al., 2014; Gombatto et al., 2015; Müller et al., 2015). In terms of walking on a treadmill, inconsistent results are reported. In terms of walking on a walkway, no study found a significant difference between groups. Participants' age did not systematically differ between studies finding and not finding a difference. The duration of chronic pain does not differ systematically with different results. Since the majority of studies including two high quality studies reported no difference, we conclude that there is moderate evidence of no differences between groups in terms of stride length, at least when walking on a walkway.

Another stride parameter examined in three studies is stride duration. In two studies, shorter stride duration in LBP group was indicated (Vogt et al., 2001, 2003). One study had high quality. A third study did not find a difference (van den Hoorn et al., 2012). All examinations were performed on a treadmill. In all studies, the average age of participants and chronicity of pain were similar. None of these confounders systematically varied. From this, we deduce that there is a tendency for shorter stride duration in LBP compared to controls since the study quality of papers reporting these results is higher. However, further studies are necessary to confirm this result.

A further three studies reported GRF. Two studies found lower GRF during walking among individuals with LBP (Zahraee et al., 2014; Müller et al., 2015). One of them is of high quality (Zahraee et al., 2014; Müller et al., 2015). The third study (Lee et al., 2007) did not find differences between people with LBP compared to controls in GRF. All studies used a walkway with a force platform to collect data. The average age of LBP participants and the definition of LBP did not systematically differ. The results provide a tendency to lower GRF especially during the push-off phase among patients with LBP compared to controls, since one study shows that this result is of high quality. Due to the limited number of studies, further studies are needed.

Looking at kinematics, there are ten studies considering rotational amplitudes during gait. Six articles found lower rotational amplitudes of the pelvis, the hip or the lumbar spine (Seay et al., 2011a; van den Hoorn et al., 2012; Crosbie et al., 2013; Gombatto et al., 2015; Müller et al., 2015; Christe et al., 2017) during walking among persons with LBP. Four other articles (Lamoth et al., 2002, 2006b; Vogt et al., 2003; Prins et al., 2016) did not report any differences. Besides one study (Müller et al., 2015), all experiments were performed on a treadmill. Participants' average age was similar in all studies. However, confounders gait conditions—like participants' age and chronicity of pain—do not explain different results. Out of the six studies showing a difference, four found a difference in pelvis rotational amplitude (Seay et al., 2011a; van den Hoorn et al., 2012; Crosbie et al., 2013; Müller et al., 2015) and two in the rotational amplitude of the lumbar spine (Gombatto et al., 2015; Christe et al., 2017). Out of the four articles reporting no difference, three investigated pelvis rotational amplitude (Lamoth et al., 2002, 2006b; Prins et al., 2016) and one hip rotation (Vogt et al., 2003). Overall, we see a tendency to reduced rotational amplitudes among persons with non-specific LBP compared to controls.

Ten studies considered the movement ratio of the lumbar spine and pelvis, which means that they looked at coordination patterns. If lumbar spine and pelvis move in the same direction, it is referred to as in-phase coordination, while if they move in the opposite direction it is referred to as anti-phase coordination. Out of these 10 studies, there is only one that could not find any difference in the movement ratio of the lumbar spine and pelvis (Vogt et al., 2001). The other studies reported more in-phase coordination for persons with LBP. There were no systematical differences in walking condition, participants' age and chronicity of pain between studies finding and the one not finding a difference. Out of the nine studies finding a difference, eight found more in-phase among persons with LBP (Selles et al., 2001; Lamoth et al., 2002, 2006a,b; Seay et al., 2011a,b, 2014; Crosbie et al., 2013). Three of the nine studies (Seay et al., 2011a,b, 2014) found a more in-phase in the frontal plane and one high quality study found more in-phase coordination in the sagittal plane (Ebrahimi et al., 2017). In three of these studies, the differences only became significant with higher demands like running (Seay et al., 2011a,b) or carrying something (Ebrahimi et al., 2017). Nonetheless, the results for more in-phase in the transverse plane are consistent. Therefore, we see moderate evidence of more in-phase coordination among subjects with LBP.

We found eight studies considering the variability of different kinematic parameters. Five of them found stronger variability among patients with LBP. Three of these five studies found a higher stride-to-stride variability of kinematic pattern (Vogt et al., 2001; Hamacher et al., 2014, 2016). Besides, the other two articles reported stronger variability in pelvis and thorax rotations LBP (Lamoth et al., 2006b) and the increase in variability with large speed changes is higher among persons with LBP (Lamoth et al., 2006a). Furthermore, one study found a reduced pelvis–trunk continuous relative phase (CRP) variability (Seay et al., 2011b) during running in the transverse plane. Two studies could not find a differences between groups, one (Seay et al., 2014) in CRP variability during running considering only one segment and the other one (Ebrahimi et al., 2017) in thigh-shank and shank foot coordination. Owing to the consistency of the results for higher stride-to-stride variability among subjects with LBP, there is moderate evidence of these results.

#### Results of neuromuscular data

Nine studies (Lamoth et al., 2006a,b; Poosapadi Arjunan et al., 2010; van der Hulst et al., 2010a,b; Hanada et al., 2011; Pakzad et al., 2016; Kim et al., 2017; Manciopi et al., 2017) considered muscle activity, whereby eight of them investigated the erector spinae (ES). Six of the eight studies reported a higher activity level for persons with LBP (Lamoth et al., 2006b; van der Hulst et al., 2010a,b; Hanada et al., 2011; Pakzad et al., 2016; Manciopi et al., 2017), whereas two had high quality. Of the other two studies, one (Poosapadi Arjunan et al., 2010) did not find a difference and another one (Lamoth et al., 2006a) reported less activity in velocity changes. With the exception of one study (Manciopi et al., 2017), all tests were performed on a treadmill. Participants' age and pain duration were similar in the studies. Therefore, we conclude that there is moderate evidence of a higher activity in ES. Out of the nine studies looking at muscle activity, three considered abdominal muscles. Two studies (Hanada et al., 2011; Kim et al., 2017) found a reduced muscle activity of abdominal muscles and one high quality study (van der Hulst et al., 2010a) found higher muscle activity in abdominal muscles. Walking condition, participants' age and pain duration did not systematically differ with the different results. In summary, there is conflicting evidence of the activation of abdominal muscles.

Variance and variability of EMG data is another parameter reported in two studies. Both showed a reduced variance or variability (Poosapadi Arjunan et al., 2010; Pakzad et al., 2016). One (Pakzad et al., 2016) found a reduced variability of trunk muscles activation among individuals with CLBP who also had high scores on the Pain Catastrophizing Scale compared to the control group. The other one (Poosapadi Arjunan et al., 2010) demonstrated that the variance of EMG amplitude during running is lower among the LBP group than in the control group. All studies performed their tests on a treadmill. These studies provide an indication for possible differences between groups at a higher level, but since there is only one study investigating each parameter further studies are needed to prove the results.

In addition, the EMG onset of hip and trunk muscles was examined in a study of high quality during walking on a treadmill. In the LBP group, the activity of hip extensors started earlier and lasted longer (Vogt et al., 2003). Because there is only one study considering the EMG onset, further studies are needed to draw conclusions.

## Discussion

The purpose this review is to provide an overview of differences between persons with and without chronic non-specific LBP in gait. The majority of included studies show moderate quality, while only a few high quality studies could be found. We identified several differences in motor control during walking. Regarding biomechanical patterns, we found at least a tendency for reduced preferred walking velocity on treadmill, a lower movement amplitude of the pelvis, more in-phase coordination, lower GRF and a higher stride-to-stride variability among the LBP group. For EMG data, we found moderate evidence of higher activity in ES among the LBP group. These results are discussed in the following.

Summarizing the results of the included case control studies on kinematic parameters, we confirm the results of the review of Laird et al. (2014) for walking. A slower movement velocity and a reduced range of movement in the hip-pelvis-lumbar region—as Laird et al. (2014) reported for certain trunk movements—are also evident in gait. Stiffening the hip-pelvis-lumbar region through higher muscular activity reduces movement in the hip and trunk joints. On the one hand, reducing movement helps to avoid further stressing the painful area. On the other hand, the continuous activity and the lack of movement can lead to the accumulation of metabolic waste products, which can be one reason for the chronification of pain (van Dieën et al., 2003). These results support the theory proposed by Hodges and Tucker (2011). In this theory, adaption to pain is seen as a protective strategy that can have negative consequences in the long term.

Regarding EMG activity, our review confirms the results of Ghamkhar et al. (Ghamkhar and Kahlaee, 2015), inasmuch as ES activity is higher among persons with chronic LBP, even though they also included studies with a specific reason for back pain. Looking at the function of ES, higher activity seems to be a response to pain. Increased activity helps to stabilize the spine and inhibit further stress on noxious structures (van Dieën et al., 2003). Additionally, we found that alterations in ES activity to changes in velocity are less adaptive among the LBP group (Lamoth et al., 2006a). Due to a high activation in rest, the normal increase of activity—as an adaption to stress—is limited. Extreme tension could be avoided and thereby the strain to musculature is reduced. As our results demonstrate, a normal adaption of muscle activity to the situations is limited, rather than having higher overall activity in trunk muscles among persons with LBP, as Ghamkhar et al. (Ghamkhar and Kahlaee, 2015) reported. Since we only found a few studies concerning the activity of other trunk muscles with inconsistent results, we can only affirm results for the ES. In order to investigate the adaptability to higher demands, further studies are needed.

As Hodges and Tucker (2011) propose, there is a causal relation between neuromuscular activity and motor outcome to create a protective mechanism. Motion between pelvis and trunk can be restricted by high activity in trunk muscles. Thus, higher activation can result in more in-phase and stiffened joints. More in-phase coordination and stiffened hip and spine joints can result in a slower preferred walking velocity due to restricted coordination. Slowing down or shortening the step length are mechanisms to minimize GRF. These are protective mechanisms that keep stress on structures low. Besides, for healthy adults there is a normal shift toward more anti-phases with increasing speed (Selles et al., 2001). Situations like adapting to a higher velocity challenge the neuromuscular system. The adaptability to different situations is one function of motor control. This function is impaired with stiffened joints and already-increased ES activity in individuals with LBP. Restricted motor control is also evident through the higher stride-to stride variability. Less compensation mechanisms lead to the higher variability in the resulting movement (Stergiou and Decker, 2011). In general, our results attest that gait control is affected among persons with non-specific LBP in the way that Hodges and Tucker (2011) propose. Furthermore, some studies provide a hint that possibly differences become more apparent in situations with higher demands like walking while carrying something (Kim et al., 2013) or solving a cognitive task (Hamacher et al., 2016).

Nonetheless, there are limitations to our findings, including the fact that the sample characteristics and survey methods differed between studies. For example, inconsistent criteria for determining non-specific LBP, different acquisition times and number of trials, different filters and different normalization methods were used in the included studies. Since non-specific pain was only defined by the exclusion of specific diseases and these exclusion criteria were controlled in different ways, we do not know for certain whether there are no subjects with a specific reason for pain included in the studies. Additionally, if only one period of back pain has a long-term influence on movement, this means that altered movement patterns persist after the pain is resolved. Consequently, subjects may have falsely been included in the control group. One study (Seay et al., 2011a) also investigates persons with LBP in a phase where their pain was resolved. In this study, motor control was still altered after the pain was resolved. By considering sample characteristics and acquisition methods in the quality assessment as well as mentioning the main confounders in the results, we tried to specify our findings. Clear standards of criteria determining LBP and a standardization of acquisition and processing methods would help to improve the comparability of studies. The usage of NOS for a methodological quality assessment led to another problem, namely we have to admit that the reporting on key criteria was incomplete and that some criteria were not mentioned in any of the studies. Therefore, it seems to be an ideal conception that is not fulfilled by the studies. Perhaps the standards of describing the methodological approach have to be modified. In our evaluation of the study quality, we made conservative estimates of the quality, which means that there were no points awarded when criteria were not reported or not fulfilled. Consequently, the methodological quality might be underestimated due to insufficient reporting rather than a poor study design or a poor methodological approach. Overall, a verification of the model proposed by Hodges and Tucker (Hodges and Tucker, 2011) and its causal relation between motor control and LBP is not possible with case control studies. Accordingly, long-term studies are necessary.

It can be summarized that there are differences in motor control between individuals with and without non-specific back pain. Further research should aim to clarify differences in motor control during tasks with higher demands where motor control is even more challenging. Furthermore, it would be interesting to conduct studies using pattern analysis to combine neuromuscular and kinematic data in the analysis. Thereby, the relation between these two levels of motor control could be better understood. For the future, the development of a motor control training using the knowledge of the altered parameters of motor control to conquer LBP effectively would be helpful. If a change of the altered parameters leads to a reduction of the development and chronification of LBP, this training will be beneficial to many people.

## Conclusion

Persons with and without non-specific LBP differ in some parameters of motor control. Despite no high evidence for any of the parameters alone, the findings match if we combine the results of measurements of the neuromuscular system via EMG and kinematic measurements. An altered motor control is identified among persons with non-specific back pain, which appears in less rotational amplitudes, a more in-phase coordination and a higher activity in ES in the LBP group.

## Author contributions

CK and FH made substantial contributions to (1) the conception, the analysis, and interpretation of data, (2) drafting, and revising it critically for important content.

### Conflict of interest statement

The authors declare that the research was conducted in the absence of any commercial or financial relationships that could be construed as a potential conflict of interest.
