# Supplementary material for: Chronic Non-specific Low Back Pain and Motor Control During Gait
Source: Front Psychol. 2018 Nov 23;9:2236. doi: 10.3389/fpsyg.2018.02236 (PMC6265306; doi:10.3389/fpsyg.2018.02236)
Supplement: Supplementary file 1 [file Table_1.DOCX]

Supplementary Material

Non-specific low back pain and motor control during gait - A systematic review

Cathrin Koch*, Frank Hänsel

*** Correspondence:** Cathrin Koch: koch@sport.tu-darmstadt.de

## Supplementary Tables

Table 1: Data extraction of included studies

| Author; year | Subjects | Condition | Variables | Findings |
| --- | --- | --- | --- | --- |
| Christe *et al.* [31] | 10 CLBP (38.7years; 4 female)  11 controls (36.7 years, 5 female) matched to age, sex and BMI | Walking across a 10m-walkway for three times at self-selected speed | Self-selected walking speed ODI, TSK, NPRS, spinal joint angles, | No sig. differences in walking speed; sig. lower range of motion in the lower lumbar spine in frontal plane; CLBP walked with sig. smaller maximal lower thoracic spine angle in transverse plane; no other sig. differences in peak angles; |
| Crosbie *et al.* [34] | 19 recurrent back pain (34,0 years, 12 female); 19 healthy controls (28,6 years, 13 female) | walking over a level walkway of 8.5-m length at preferred speed and at as fast a speed as was comfortable, 30 sec rest between trials | ROM, angular displacements, movement coordination, phase lag | LBP group had decreased pelvic side flexion and hip flexion at all time points but greater pelvic axial rotation at heel strike; In LBP group side flexion and axial rotation had a near linear relationship; Phase lag for axial rotation was sig. less in LBP than in controls at a preferred speed |
| Ebrahimi *et al.* [22] | 10 CLBP (29.8 years, 5 female);  10 controls (29.6 years, 5 female) matched according to sex, age, weight and height | Walking barefoot along a 8m-walkway for 5 to 6 times | Preferred walking speed; intersegmental coordination (trunk-pelvis, lower extremities) | No sig. differences in preferred walking speed; sig. more trunk-pelvis in-phase coordination in sagittal plane in CLBP; no sig differences in pelvis-thigh coordination; left thigh-shank and shank-foot mean absolute relative phase were more in-phase in CLBP; no differences in thigh-shank or shank-foot coordination variability |
| Gombatto *et al.* [23] | 18 LBP (28,1 years, 11 female); 18 controls (27,6 years; 8male) | min of 3 trials walking along a 10m track with pacing steps to the beats of a metronome with a target pace of 1.2 m/sec | kinematics of the upper and lower lumbar spine; gait parameters (speed, step length, stride length, cycle time) | LBP group displayed less rotation in axial plane; no group effect for coronal and sagittal plane; no differences in gait parameters |
| Hamacher *et al.* [35] | 12 CLBP (51 years); 12 controls (51 years) | only cognitive task, only walking on a 25m walkway for two minutes, 3min break, then combination of walking and cognitive task | stride to stride variability, dual task costs | group by condition interaction effect for gait variability, group effect for gait variability in dual task condition with a higher variability for LBP group; dual task cost higher in LBP, no difference in control group |
| Hamacher *et al.* [41] | 12 CLBP (57 years);  12 controls (55 years) | Walking along a 25m-walkway for 2 min at self-selected speed; walking for 2 min simultaneously performing the Regensburger word fluency test | stride to stride variability, stride length, minimum toe clearance | Variability of stride length in CLBP sig. higher in dual task, while the increase was not sig. in controls; sig. decreased stride time variability in CLBP and controls; no sig differences in minimum toe clearance; CLBP sig. higher stride time variability in single and dual task walking |
| Hanada *et al.* [17] | 9 LBP (61.4 years; 5 female); 9 controls (64.9 years; 5 female) | 4 trials of walking at a self-selected speed over a GAITRite mat | base of support, speed, stride length, EMG amplitudes | LBP group walked with a wider base of support, none of the other gait parameters are different; lower activation of left and right RA in LBP; LBP higher activation of left LES than controls |
| Kim *et al.* [32] | 30 LBP (15 low intensity LBP (22.6 years, 6 female), 15 high intensity LBP (23.5 years, 6 female));  15 controls (22.5 years, 6 female) matched to age and sex | Walking along a walkway at self-selected speed for 12 gait cycles | Average muscle activity for RA, EO,IO | Sig. decreased right IO activity in both groups of LBP; no sig difference in abdominal activity between the groups with LBP |
| Lamoth *et al.* [36] | 39 LBP (38 years, 27female);  19 controls (41 years, female) | familiarization on treadmill; velocity increased by 0.8km/h starting with 1.4km/h up to 5.4km/h; data acquisition for 30 sec | velocity, ROM, index of harmonicity (HI), continuous relative Fourier phase (RFP), weighted coherence | walking velocity lower in LBP; no difference in ROM; HI lower in LBP at 5.4 km/h; The RFP mean increased with increasing walking velocity and was higher in the control group than in the LBP group; at low walking velocities, the coupling between pelvis and thorax was stronger in the LBP group than in the control group |
| Lamoth *et al.* [26] | 19 CLBP ( 38 years, 11 female); 14 controls (31 years, 5 female) | warm-up on treadmill, walking at preferred speed, walking at speeds of 1,4 up to 7,0 km/h , with increments of 0,8 Km/h; | comfortable walking velocity, stride length, rotational amplitudes, continuous relative phase, mean EMG | comfortable walking velocity was lower in the LBP group; stride length was shorter in LBP at 1.4, 2.2, 3.0 und 6.2 km/h; no differences in rotational amplitudes for transverse and frontal plane; global pattern of thoracic, lumbar and pelvic rotations was highly consistent across all participants and velocities; variability of the residual patterns of transverse thoracic and lumbar rotations was smaller in the LBP participants than in controls at comfortable velocity; variability of transverse lumbar rotations were smaller in the LBP participants than in controls at prescribed velocities; mean amplitude of ES higher in during swing phase; |
| Lamoth *et al.* [25] | 12 LBP (26.8 years, 7 female); 12 controls (30 years, 5 female) | warm-up on treadmill, walking at preferred speed, recordings were then performed at six velocities in a fixed order: 6.2, 1.4, 3.8, 5.4, 2.2, and 4.6 km/h | comfortable walking velocity, stride length, relative phases between trunk segments, patterns of trunk coordination, ES activity, | comfortable walking velocity was lower in the LBP group; stride length was shorter in LBP at 6,2 km/h; at the three highest velocities the change towards antiphase coordination of Rpthorax-pelvis (thpe) was reduced in the LBP group, RP lumbar spine-pelvis stable; no effect in frontal plane; SD RPthpe und RPlupe were in LBP group, increase with higher velocities was higher in LBP; no differences in global pattern of trunk coordination; compared to the control group, residual variability was smaller in the LBP group for transverse plane rotations and larger for frontal plane rotations at all velocities; no differences in global pattern LES activation; maximal activation of LES earlier at increasing speed in LBP |
| Lee *et al.* [27] | 20 LBP only (46.0 years);  20 controls (46.4 years); matched to age (+/-5 years) and sex | 3 trials of walking on an elevated walkway with preferred and fastest walking speed, last trail was used for analysis | gait velocity; vertical ground reaction forces | LBP slower than the controls at preferred walking speed, but groups had comparable normalized gait velocity during their fastest walking speed condition; LBP and control groups have no difference for all vertical GRF parameters during both walking speed conditions when gait velocity was taken into account |
| Manciopi *et al.* [42] | 15 CLBP (31.9 years, 9 female);  15 controls (30.3 years, 9 female) | Gait (barefoot) combined with object prehension; walk-through without grasping | Coactivation-index of ES, dynamic stability, gait phases | In the combined task reduced walking speed, increased EMG activity and increased width of base of support in LBP |
| Müller *et al.* [37] | 11 CLBP (38,2 years; 6 female),  11 controls (38,5 years; 6 female); matched to age, sex, height, weight | walking on a 17 m walkway with force platform at self-selected velocity, first walking then running; afterwards the setup was changed, a force platform elevated at initial contact; again first walking then running until 5 successful trials of every condition were recorded | Gait velocity, stride length, ground reaction forces, rotational amplitudes in transversal plane, in sagittal plan trunk inclination, knee and ankle angles | velocity lower in LBP on even and uneven ground; no difference in stride length; on even ground decrease of early GRF in LBP; on uneven ground lower GRF on first and second contact; amplitude of pelvis rotation lower on even and uneven ground; during running on uneven ground in LBP patients peak ground reaction force at elevated first contact decreased as compared to controls; in LBP knee joint at the lowered second contact was more extended at touchdown as compared to the controls |
| Newell and van der Laan [30] | 12 CLBP (28 years; 7 female); 12 controls (27 years; 8 female) | Walking on treadmill; finding preferred walking speed by increasing velocity; than decreasing to preferred speed; and increasing again; average of three values; walking 8 min barefoot or on socks | Preferred walking speed; stride length; Fractal scaling index for each foot | Fractal scaling index for the left foot was lower in LBP; no other sig. results |
| Pakzad *et al.* [33] | 30 CLBP (15 high pain catastrophizing (34.1 years; 9 female) 15 low pain catastrophizing (32.5 years, 9 female);  15 controls (33.1 years, 9 female) matched to age and sex | Walking on treadmill with self-selected speed for at least 20 gait cycles | EMG (RA,EO,ES,LM) | High pain catastrophizing LBP had a significant higher EMG amplitude than controls in LM and right RA; reduced variability of muscle activation in HLBP |
| Poosapadi Arjunan *et al.* [15] | 4 LBP ( 39 years, 4 male); 9 controls (29. 8 years, 9 male) | 10 min walking (4km/h) and running (8km/h) on treadmill, 15 min break in between, it was allowed to stop if | variance of amplitude, change in the variance of amplitude | no difference in the variance of the amplitude or in the change of variance over time of sEMG of the two cohorts in walking; increase in the variance for both cohorts when the participants were running, the increase in the LBP patients was much greater when compared with the healthy participants |
| Prins *et al.* [20] | 15 CLBP (34 years, 15 male);  15 controls (33 years, 15 male) | Walking on treadmill at 3.8 km/h; 1. No perturbation, 2. 2 min Perturbation trial, 3. 5 min perturbation trial | Segmental rotation | No sig. differences in segmental rotation in unperturbed and perturbed gait |
| Seay *et al.* [28] | 14 LBP: 14 (35,71 years, 6 female); 14 RES ( 32,56 years, 5 female);  14 controls (29,9 years, 8 female) | 10 min warm-up on treadmill; determination of preferred walking speed; then start at 0,8m/s with increasing speed every 30 s for 0,5 m/sup to 3,8m/s, data acquisition in the last 20 sec | Gait velocity, ROM over at least 10 steps; coordination (relative movements of spine and pelvis) | no difference in preferred walking velocity; greater axial rotation in LBP group during running compared to control group and nearly compared to resolved group; group x velocity-interaction, LBP show higher increase in rotations than the others; walking the LBP group spent more time in-phase in lateral flexion than the control group, during running LBP group spent more time in in-phase coordination in the transverse plane |
| Seay *et al.* [39] | 14 LBP: 14 (35,71 years, 6 female); 14 RES ( 32,56 years, 5 female);  14 controls (29,9 years, 8 female) | 10 min warm-up, running at 2,3m/s, increasing by steps of 0,5 m/s up to 3,8 m/s; every 30 sec., data acquisition in the last 20 sec | CRP, CRP variability | LBP group showed more in-phase coordination in frontal plane in walking mode; LBP group showed more in-phase coordination than the RES group but not sig.; LBP group showed more in-phase compared to the RES and control group in transverse plane during running; control group demonstrated increased CRP variability as compared to the LBP group during running |
| Seay *et al.* [38] | 14 LBP: 14 (35,71 years, 6 female); 14 RES ( 32,56 years, 5 female);  14 controls (29,9 years, 8 female) | 10 min warm-up, running at 2,3m/s, increasing by steps of 0,5 m/s up to 3,8 m/s; every 30 sec., data acquisition in the last 20 sec | CRP, CRP variability | bend–twist CRP was lower for the LBP group than controls regardless of running speed; no difference in CRP variability |
| Selles *et al.* [29] | 6 LBP (30 years; 4 female);  6 controls (30 years; 2 female) | Familiarization on treadmill; 1 min comfortable walking speed; increasing velocity from 0.17 to 1.5 m/s in steps of 0.22 m/s, then decreasing in similar steps; data collection for 30 sec each step | Comfortable velocity, movement coordination, Left-right symmetry | Reduced comfortable walking speed in LBP; 4 out of 6 patients were not able to establish out-of-phase coordination pattern at higher speeds; increased stability in these patients; increased asymmetry of phase relations of left and right side in LBP group |
| van den Hoorn *et al.* [40] | 13 LBP ( 35.3 years, 8 female); 12 controls (32.2years, 8 female) | walking on treadmill, 3min at 12 levels from 0,5 to 1,72 m/s with an increments of 0.11 m/s; 2 min adaption to velocity, then 1 min data recording, after 5 levels 5 min break | stride length, stride time, walking velocity, segmental rotations | no differences in stride length and time; lower preferred walking velocity in LBP; mean absolute residual rotations of pelvis or thorax did not differ between the Groups; mean absolute trunk residual rotations differed between groups; LBP participants had smaller absolute relative residual rotations; Pearson correlations between pelvis and thorax residual rotations were higher in LBP |
| van der Hulst *et al.* [16] | 96 out 123, 63 CLBP (41 years; 8 female), 33 controls (40 years; 17 female); matched to age, sex and BMI; due to technical problems only 91 subjects analyzed (59 CLBP, 32 controls) | 5 min familiarization on treadmill; speed of 1,4 up to 5,4 km/h , with increments of 0,8 Km/h; 2 min between trials to check data for at least 20 steps | SRE (smooth rectified EMG) (ES/RA/EO) | Averaged SRE values of ES were 1.20 times higher in subjects with CLBP when compared to controls; Averaged SRE values of RA were on average 1.36 times higher in subjects with CLBP than controls during the total stride; average SRE values of OE were comparable between subjects with CLBP and controls |
| van der Hulst *et al.* [21] | 96 out 123, 63 CLBP (41 years; 8 female), 33 controls (40 years; 17 female); matched to age, sex and BMI; due to technical problems only 91 subjects analyzed (59 CLBP, 32 controls) | 5 min familiarization on treadmill; speed of 1,4 up to 5,4 km/h , with increments of 0,8 Km/h; 2 min between trials to check data for at least 20 steps | SRE (smooth rectified EMG) (ES/RA/EO); duration of stride phase and stance phase | averaged SRE values of ES were higher in LBP compared with controls, otherwise comparable pattern; duration of the stride phase was comparable between both groups as well as in stance phase |
| Vogt *et al.* [19] | CLBP:34 ( 34,2 years, 13 female); 22 Controls (32,1 years, 6 female) | familiarization on treadmill; then 3 min of walking at 4.5 km/h, data acquisition for 30 sec | cycle duration, angular displacement, stride-to-stride variability | cycle duration shorter in CLBP compared to controls; similar oscillation pattern in both groups in all planes; higher stride-to stride variability in CLBP in each of the cardinal planes |
| Vogt *et al.* [24] | 17 CLBP (36.3 +/-2.1 years, 17 male); 16 controls (33.7 years,16 male); matched to age | practice treadmill walking; 15 min break, walking again until they feel comfortable on treadmill at 1.25 m/s, data acquisition over a minimum of 20 strides | ROM, stride time, EMG activity (onset, pattern) | lower hip joint range of motion and shorter stride time in back pain patients compared to controls; differences in EMG-onset of hip extensors and lumbar ES, earlier and longer activity in LBP; almost identical patterns of falling and rising trends in cross-correlation pattern |
| Zahraee *et al.* [18] | 20 LBP (41.56 years, 20 female); 20 controls (40.18 years, 20 female); matched to age and height | 5 trials walking along gait lab path with comfortable walking speed | Gait velocity (cm/s), cadence (steps/min), step length (cm), maxima of vertical forces, medio-lateral forces, anterior-posterior forces, asymmetry index measure | no difference in spatio-temporal gait parameters; higher vertical forces in controls; no differences for medio-lateral or anterior-posterior forces between groups; no difference for ASI |

CLBP chronic low back pain; RES resolved back pain group; RMS root mean square; GRF ground reaction forces; ROM range of motion; (C)RP (continuous) relative phase; (L)ES m. erector spinae (lumbar); RA m. rectus abdominis; OE m. obliquus externus; ASI asymmetry index
