# Supplementary material for: Chronic Non-specific Low Back Pain and Motor Control During Gait
Source: Front Psychol. 2018 Nov 23;9:2236. doi: 10.3389/fpsyg.2018.02236 (PMC6265306; doi:10.3389/fpsyg.2018.02236)
Supplement: Supplementary file 2 [file Table_2.DOCX]

Supplementary Material

Non-specific low back pain and motor control during gait - A systematic review

Cathrin Koch*, Frank Hänsel

*** Correspondence:** Cathrin Koch: [koch@sport.tu-darmstadt.de](mailto:koch@sport.tu-darmstadt.de)

**Table 2: Quality assessment of included studies**

| Study | 1 | 2 | 3 | 4 | 5 | 6 | 7 | 8 | 9 | 10 | Score |
| --- | --- | --- | --- | --- | --- | --- | --- | --- | --- | --- | --- |
| Christe *et al.* [31] | 1 | k | k | 1 | 2 | 1 | 1 | k | 1 | k | 7 |
| Crosbie *et al.* [34] | 2 | k | k | 0 | 0 | 1 | 1 | k | 1 | k | 5 |
| Ebrahimi *et al.* [22] | 2 | k | 1 | 1 | 2 | 1 | 1 | k | 1 | k | 9 |
| Gombatto *et al.* [23] | 1 | 0 | 1 | 1 | 2 | 1 | 1 | k | 1 | k | 8 |
| Hamacher *et al.* [35] | 0 | k | 1 | k | 1 | 1 | 1 | k | 1 | k | 5 |
| Hamacher *et al.* [41] | 1 | k | 1 | k | 0 | 1 | 1 | k | 1 | k | 5 |
| Hanada *et al.* [17] | 1 | 0 | 1 | k | k | 1 | 1 | k | 1 | k | 5 |
| Kim *et al.* [32] | 0 | k | 1 | k | 2 | 1 | 1 | k | 1 | k | 6 |
| Lamoth *et al.* [36] | 1 | 0 | k | 1 | 0 | 1 | 1 | k | 1 | k | 5 |
| Lamoth *et al.* [26] | 2 | 0 | 1 | 1 | 0 | 1 | 1 | k | 1 | k | 7 |
| Lamoth *et al.* [25] | 2 | k | k | 1 | 0 | 1 | 1 | k | 1 | k | 6 |
| Lee *et al.* [27] | 1 | 0 | k | 0 | 2 | 1 | 1 | k | 1 | k | 6 |
| Manciopi *et al.* [42] | 1 | k | k | 1 | 0 | 1 | 1 | k | 1 | k | 5 |
| Müller *et al.* [37] | k | k | k | k | 2 | 1 | 1 | k | 1 | k | 5 |
| Newell and van der Laan [30] | 1 | k | k | 0 | 2 | 1 | 1 | k | 1 | k | 6 |
| Pakzad *et al.* [33] | 1 | k | k | 1 | 2 | 1 | 1 | k | 1 | k | 7 |
| Poosapadi Arjunan *et al.* [15] | 2 | 0 | k | 1 | 0 | 1 | 1 | k | 1 | k | 6 |
| Prins *et al.* [20] | 1 | k | 1 | 1 | 0 | 1 | 1 | k | 1 | k | 6 |
| Seay *et al.* [38] | 1 | k | k | 1 | 0 | 1 | 1 | k | 1 | k | 5 |
| Seay *et al.* [28] | 1 | 0 | 1 | 1 | 0 | 1 | 1 | k | 1 | k | 6 |
| Seay *et al.* [39] | 1 | k | k | 1 | 0 | 1 | 1 | k | 1 | k | 5 |
| Selles *et al.* [29] | 2 | k | k | 1 | k | 1 | 1 | k | 1 | k | 6 |
| van den Hoorn *et al.* [40] | 1 | 0 | 1 | 0 | 0 | 1 | 1 | k | 1 | k | 5 |
| van der Hulst *et al.* [21] | 2 | 1 | 1 | 0 | 2 | 1 | 1 | 0 | 1 | k | 9 |
| van der Hulst *et al.* [16] | 2 | 1 | 1 | 0 | 2 | 1 | 1 | 0 | 1 | k | 9 |
| Vogt *et al.* [19] | 2 | 0 | 1 | 0 | 0 | 1 | 1 | k | 1 | k | 6 |
| Vogt *et al.* [24] | 2 | 0 | 1 | 1 | 1 | 1 | 1 | k | 1 | k | 8 |
| Zahraee *et al.* [18] | 1 | 0 | 1 | 1 | 2 | 1 | 1 | k | 1 | k | 8 |

Questions: 1 adequate case definition; 2 representativeness of the cases; 3 selection of controls; 4 definition of controls; 5 comparability of cases and controls; 6 valid data acquisition; 7 reliable data acquisition; 8 blinding of investigators; 9 same method of ascertainment for cases and controls; 10 reporting of non-response rate; Evaluation: 1 “Yes”; 0 “No”; k not reported; 2 more than just standard; * Definition of control group is given, but in the analysis considered as one of six clusters
