# Supplementary material for: Chronic Non-specific Low Back Pain and Motor Control During Gait
Source: Front Psychol. 2018 Nov 23;9:2236. doi: 10.3389/fpsyg.2018.02236 (PMC6265306; doi:10.3389/fpsyg.2018.02236)
Supplement: Supplementary file 3 [file Data_Sheet_1.docx]

Supplementary Material

Non-specific low back pain and motor control during gait - A systematic review

Cathrin Koch*, Frank Hänsel

*** Correspondence:** Cathrin Koch: koch@sport.tu-darmstadt.de

# Supplementary Data

**Quality assessment score**

Selection

1. Is case definition adequate? (Max. 2 points)
2. Yes, defined by inclusion and exclusion criteria (1)
3. Yes, defined inclusion and exclusion criteria are measured with standardized and objective means (1)
4. Only based on self-reports (0)
5. No description (k)
6. Representativeness of the cases (Max. 1 point)
7. Consecutive or obviously representative series of cases (1)
8. Potential for selection biases or not stated (0)
9. No description (k)
10. Selection of controls (Max. 1 point)
11. Community controls (1)
12. Controls with another common characteristic (e.g. hospital controls) (0)
13. No description (k)
14. Definition of controls (Max. 1 point)
15. No visit to the doctor or sick day due to LBP (1)
16. Other than described in a) (0)
17. No description (k)

Comparability

1. Comparability of cases and controls on the basis of the design or analysis (Max. 2 points)
2. Study controls for age (through matching) (1)
3. Study controls for an additional factor like sex, height, weight (1)
4. No matching (0)
5. No description (k)

Acquisition

1. Is the method of data acquisition valid? (Max. 1 point)
2. Yes (1)
3. No (0)
4. Is not known (k)
5. Is the method of data acquisition reliable? (Max. 1 point)
6. Yes (1)
7. No (0)
8. Is not known (k)

Exposure

1. Was (were) the outcome assessor(s) aware of the exposure status of participants? (Max. 1 point)
2. Yes (1)
3. No (0)
4. No description (k)
5. Same method of ascertainment for cases and controls (Max. 1 point)
6. Yes (1)
7. No (0)
8. No description (k)
9. Non response rate (Max. 1 point)
10. Same rate for both groups (1)
11. Different rates (0)
12. No description (k)
